# Validation of acute physiology and chronic health evaluation (APACHE) IV score in a korea provincial icus by comparing korean simplified acute physiology score (SAPS) III

**DOI:** 10.1186/2197-425X-3-S1-A335

**Published:** 2015-10-01

**Authors:** JY Moon, IS Kwon, JW Lee, S-I Park, G-R Chon, JY Lee, J-Y Ahn, Y-J Chang, S-J Kwon, Korean Chungcheong Critical Care Research Group (KCCCRG)

**Affiliations:** Internal Medicine, Chungnam National Univ. Hospital, Daejeon, Korea Republic of Korea; Clinical Trials Center, Chungnam National Univ. Hospital, Daejeon, Korea Republic of Korea; Daejeon Regional Emergency Center, Chungnam National University Hospital, Daejeon, Korea Republic of Korea; Anesthesiology and Pain Medicine, Chungnam National University Hospital, Daejeon, Korea Republic of Korea; Chungju Hospital, Konkuk University School of Medicine, Internal Medicine, Chungju, Korea Republic of Korea; Internal Medicine, Chungbuk National Univ. Hospital, Cheongju, Korea Republic of Korea; Internal Medicine, Konyang University Hospital, Daejeon, Korea Republic of Korea

## Introduction

The Simplified Acute Physiology Score (SAPS) III has been validated as the Korea SAPSIII model in Korea. However, the Acute Physiology and Chronic Health Evaluation (APACHE) IV model has not yet been validated in Korea provincial intensive care units (ICUs).

## Objectives

The aim of this study was to compare the ability of the APACHE IV with Korean SAPS 3 in predicting hospital mortality in a provincial ICU population.

## Methods

The study was designed as a prospective cohort study for patients admitted to the nine intensive care units in the four provincial academic medical centers from September 2013 to February 2014. Validation and comparison were conducted to measure discrimination and calibration with using the area under the receiver operating characteristic curve (AUC) and the Hosmer-Lemeshow test, respectively.

## Results

Among 733 enrolled patients, 34.4% (252/733) were surgical patients. The hospital mortality was 23.5%. The median APACHE IV score was 71(Standard deviation ± 33.8), and the predicted death rate was 25.6% respectively. The observed hospital mortality was 23.5%. The discriminative powers of two models were similar. The AUCs were 0.753 (95% confidence intervals (CI) : 0.711-0.794) for APAPCH IV and 0.768 (95% CI : 0.727-0.809) for Korean SAPS III respectively. Hosmer-Lemeshow C and H statics showed good calibration for both models, (H = 8.69, p = 0.370; C = 128.17, p = 0.824 for APACHE IV, and H = 8.40, p = 0.396; C = 178.98, p < 0.001 for Korean SAPS III respectively).

## Conclusions

For Korea regional ICU patients, the APACHE IV and Korea SAPS III showed good discrimination and good calibration for hospital mortality. Therefore, the APACHE IV prognostic model might be applied to predict mortality in Korea regional ICUs.
